# Supplementary material for: Paper microfluidic implementation of loop mediated isothermal amplification for early diagnosis of hepatitis C virus
Source: Nat Commun. 2021 Nov 30;12:6994. doi: 10.1038/s41467-021-27076-z (PMC8632961; doi:10.1038/s41467-021-27076-z)
Supplement: Supplementary file 1 — Supplementary Information [file 41467_2021_27076_MOESM1_ESM.pdf]

Loop mediated isothermal amplification as a powerful tool for early diagnosis of hepatitis C virus.

Weronika Witkowska McConnell[1,2], Chris Davis[1], Suleman R. Sabir[1], Alice Garrett[2], Amanda Bradley-Stewart[3], Pawel Jajesniak[2], Julien Reboud[2], Gaolian Xu[2], Zhugen Yang[2], Rory Gunson[3], Emma C. Thomson[1*], Jonathan M. Cooper[2*]

1. MRC-University of Glasgow, Centre for Virus Research, University of Glasgow, United Kingdom.

2. Division of Biomedical Engineering, James Watt School of Engineering, University of Glasgow, United Kingdom.

3. West of Scotland Specialist Virology Centre, New Lister Building, Glasgow Royal Infirmary, United Kingdom.

*Correspondence to Emma C. Thomson (emma.thomson@glasgow.ac.uk) and Jonathan M. Cooper (jon.cooper@glasgow.ac.uk)

Supplementary Information

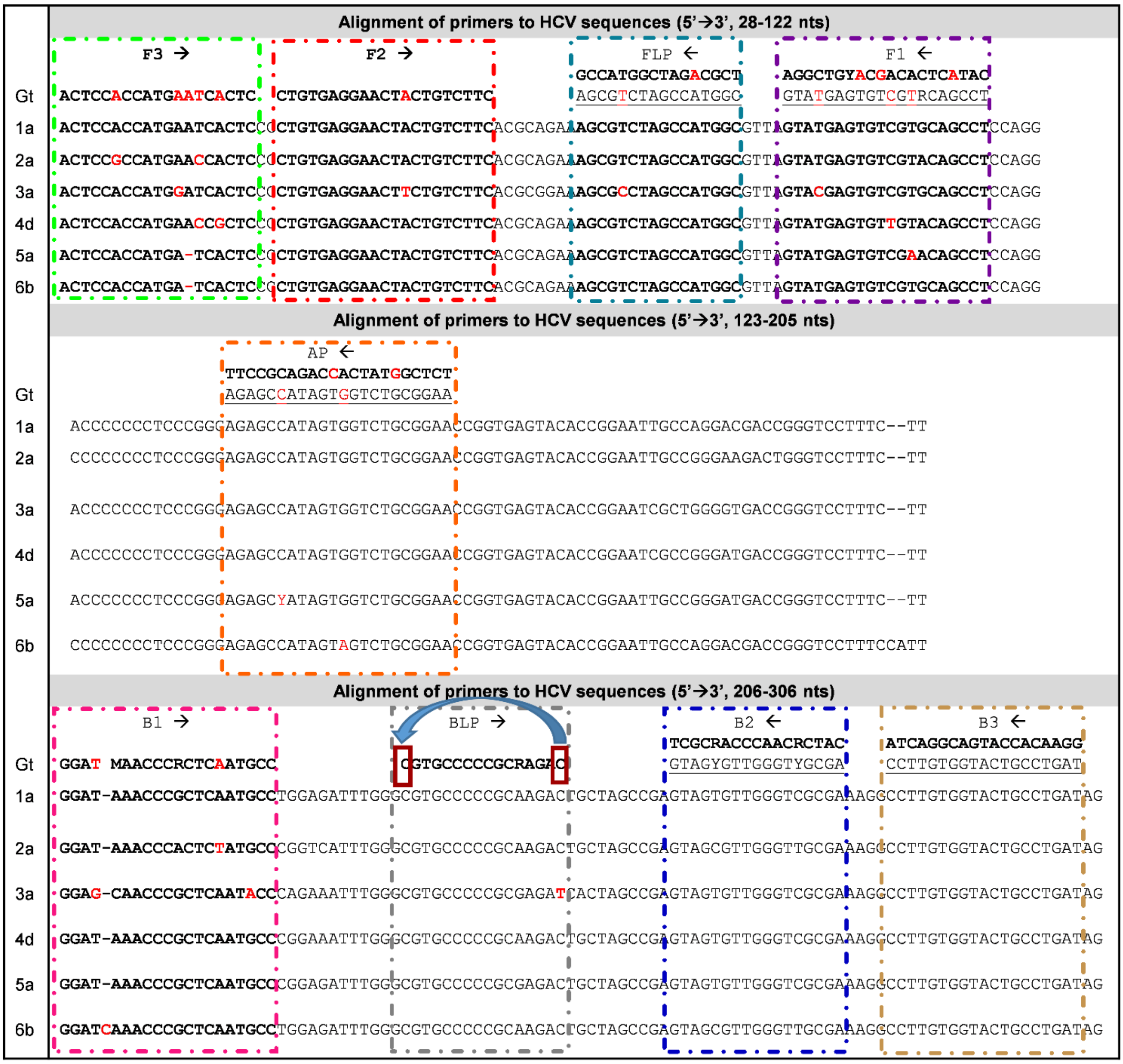

Fig. S1: Alignment of LAMP primers with six major HCV genotypes.

The alignment was performed using LAMP primers and six major HCV genotypes (Genbank: AF009606, 1a, D00944, 2a, D17763, 3a, FJ462437, 4d, KC844046, 5a, D84262, 6b). The nucleotide numbering is based on the H77 AF009606 sequence in the 5' untranslated region from nucleotides 28-306. Dashed lines indicate areas of individual primer targets. Black arrows pointing right indicate forward primers and those pointing left indicate reverse primers. For reverse primers, the underlined sequence represents the complementary primer sequence. Mismatches are indicated in red. The blue arrow and red squares indicate the cytosine change from the original backward loop primer (BLP) to the new BLP. [main text reference – Yang et al.[17]],

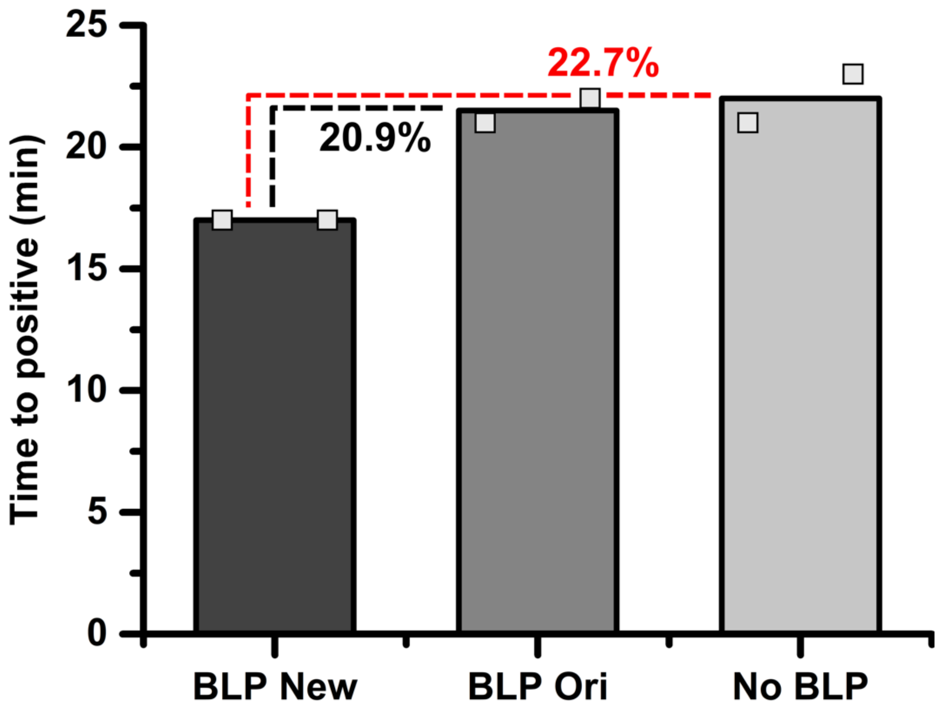

Fig. S2: Evaluation of novel backward loop primer in genotype 3 standard.

Time to positive was calculated based on LAMP assays incubated with genotype 3 standard as a positive template. All LAMP assays were incubated for 45 minutes and consisted of 0.8μM of FIP/BIP, 0.4μM AP, 0.4μM loop primers (or 0.4μM FLP only for No BLP assays) and 0.2μM of F3/B3. BLP New represents the novel BLP and BLP Ori represents the original published primer.[1] The dashed lines with numbers show the percentage decrease in time to positive of the BLP New assays from all other LAMP assays. Graph shows mean and individual points (gray squares, n=2). Source data are provided as a Source Data file.

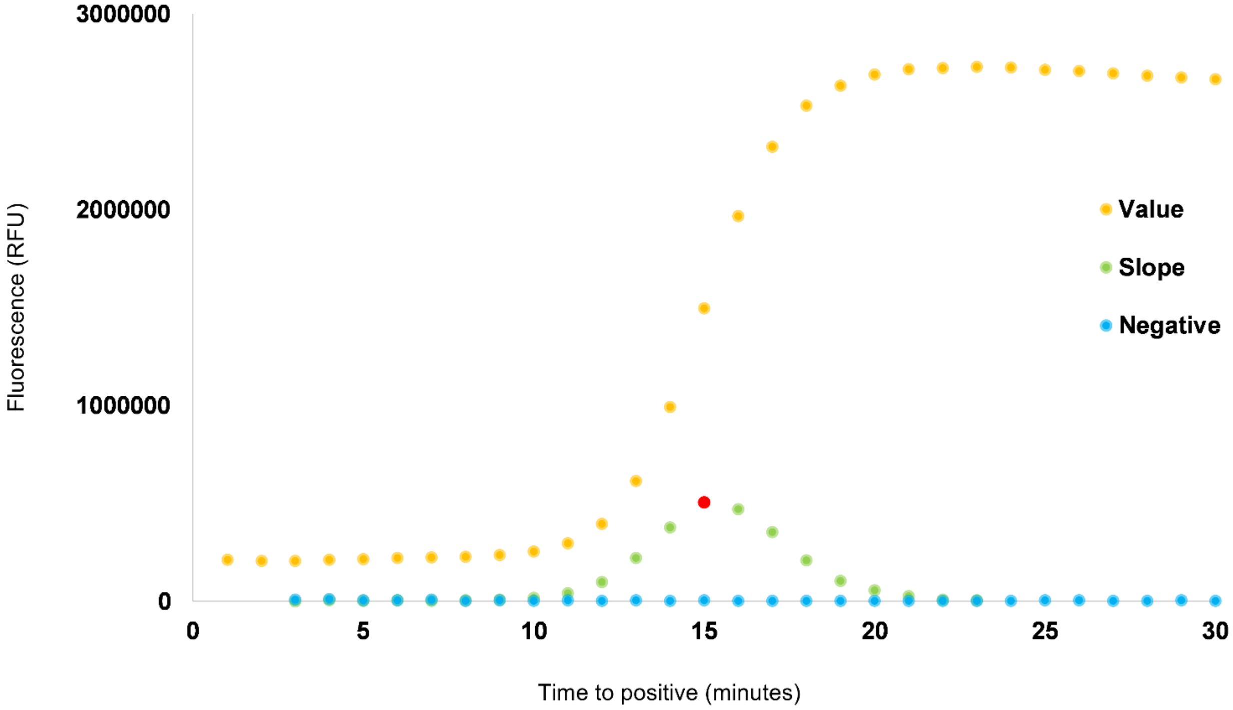

Fig. S3: Determination of time to positivity.

The value curve shows increase in relative fluorescence units (RFU) over 30-minutes at 65◦C with genotype 1a standard. The slope (yellow) was measured by subtracting fluorescence from the previous time point (a derivative). The highest point located on the slope is defined as the time to positive (shown in red). Samples with ten times standard deviation of mean baseline RFU at the time to positive point were defined as positive. The negative control (shown in blue) resulted in minimal increase in RFU over-time. Source data are provided as a Source Data file.

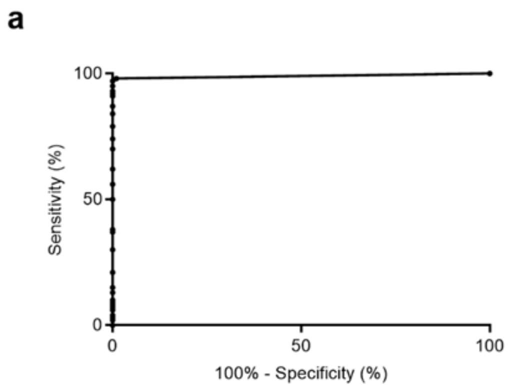

Area under the ROC curve	
Area	0.9899
Std Error	0.008186
95% CI	0.9738 to 1.006
P value	<0.0001

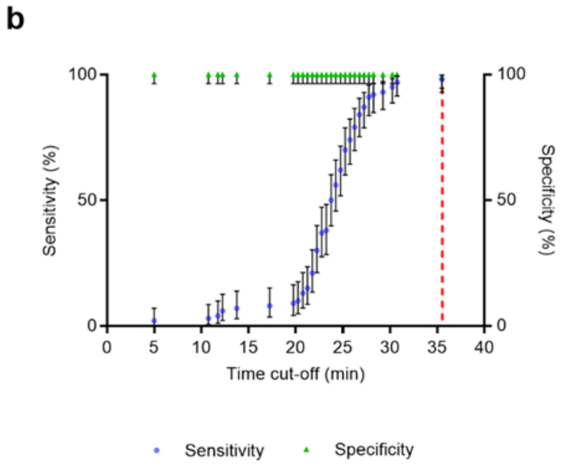

Fig. S4: ROC curve for RT-PCR and Ct cut-off determination.

ROC curves were based on mean Ct value of 100 HCV positive and 100 HCV negative samples. ROC curve demonstrates the area under the curve, standard error (Std Error), 95% confidence intervals (95% CI) and the p value. (a). ROC curve analysis for RT-PCR and cDNA samples performed using the Clopper-Pearson Method (one-sided, no adjustments for multiple comparisons). (b). Graph showing sensitivity (circle) and specificity (triangle) at different Ct values for RT-PCR. Error bars are 95% confidence intervals. The dashed line indicates the cut-off Ct value for RT-PCR, where sensitivity and specificity are the highest. Source data are provided as a Source Data file.

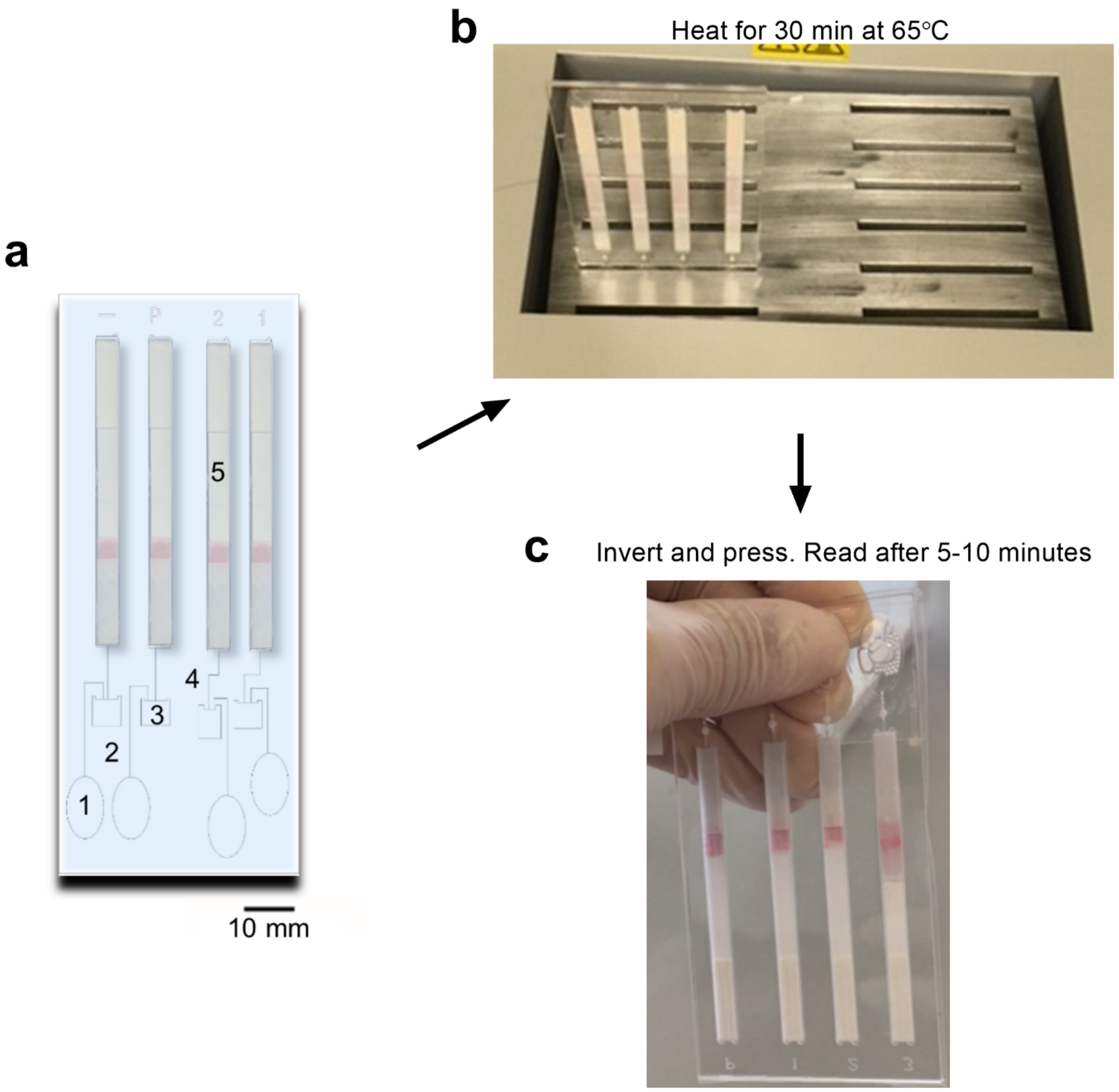

Fig. S5: Workflow for lateral flow LAMP devices.

(a). Lateral flow devices were laser cut in-house from poly (methyl methacrylate). Each 2 mm-thick device was sealed with acetate film on either side and contains an inlet(1), water chamber(1), water channel(2), LAMP chamber(3) and LAMP channels(4) connecting LAMP chambers with the nucleic acid detection strips(5). (b). After the addition of template through the inlet into the LAMP reaction, the device was sealed with acetate film and inserted upright into a heat block for 30 minutes at 65◦C. (c). Following the incubation period, the device was inverted and pressure was manually applied to each water chamber until the liquid reached the conjugate pad line. The lateral flow strips were left for 5-10 minutes and the results read subsequently.

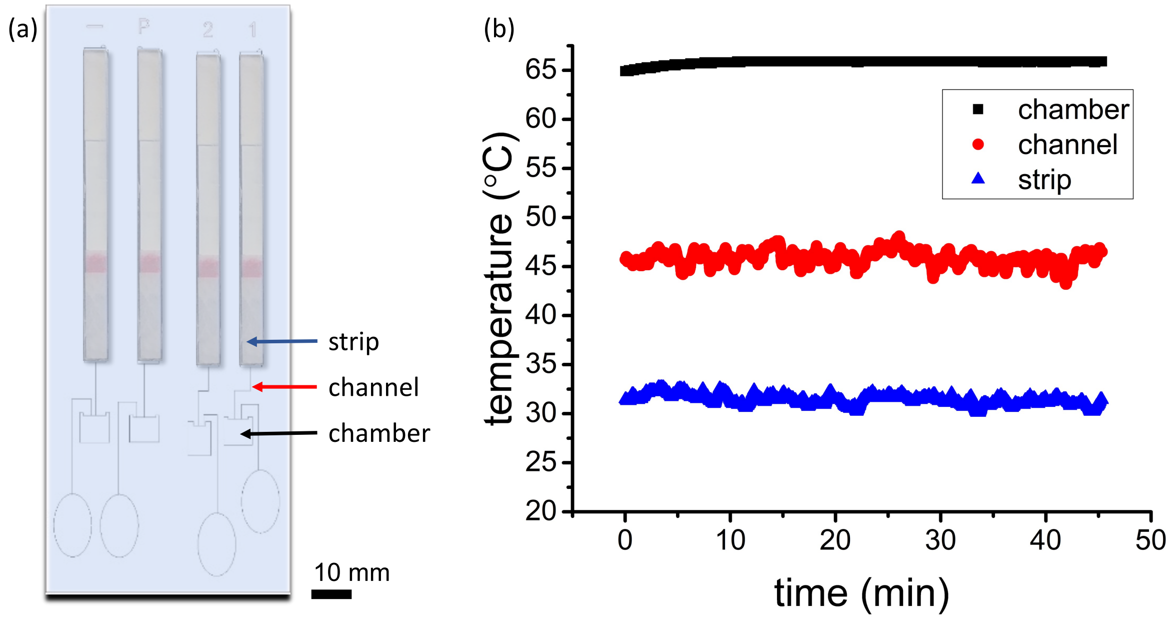

Fig. S6. Temperature monitoring during the LAMP reaction in the heater device (Fig. S5b). The temperature was measured using thermocouples placed in different positions in device (a). (b) Results show a stable temperature at the LAMP reaction value of 65.8°C (black square, +/- 0.2°C, standard deviation), whilst it decreases rapidly away from it to reach close to room temperature (which was 20°C) for measurements performed on the strips (blue triangles, 31.3°C +/- 0.7°C). The higher fluctuations for the temperatures measured in the channels (red disc) and the strip can be explained by the fact that these areas are exposed to the room airflow. Source data are provided as a Source Data file.

Table S1: Sensitivity and specificity at different Ct values for RT-PCR.

Time cut-off (minutes)	Sensitivity (%)	95% CI	Specificity (%)	95% CI
< 5	2	0.2431% to 7.038%	100	96.38% to 100%
< 10.75	3	0.623% to 8.518%	100	96.38% to 100%
< 11.75	4	1.1% to 9.926%	100	96.38% to 100%
< 12.25	6	2.233% to 12.6%	100	96.38% to 100%
< 13.75	7	2.861% to 13.89%	100	96.38% to 100%
< 17.25	8	3.517% to 15.16%	100	96.38% to 100%
< 19.75	9	4.198% to 16.4%	100	96.38% to 100%
< 20.25	10	4.9% to 17.62%	100	96.38% to 100%
< 20.75	13	7.107% to 21.2%	100	96.38% to 100%
< 21.25	15	8.645% to 23.53%	100	96.38% to 100%
< 21.75	21	13.49% to 30.29%	100	96.38% to 100%
< 22.25	30	21.24% to 39.98%	100	96.38% to 100%
< 22.75	37	27.56% to 47.24%	100	96.38% to 100%
< 23.25	38	28.48% to 48.25%	100	96.38% to 100%
< 23.75	50	39.83% to 60.17%	100	96.38% to 100%
< 24.25	56	45.72% to 65.92%	100	96.38% to 100%
< 24.75	62	51.75% to 71.52%	100	96.38% to 100%
< 25.25	70	60.02% to 78.76%	100	96.38% to 100%
< 25.75	74	64.27% to 82.26%	100	96.38% to 100%
< 26.25	79	69.71% to 86.51%	100	96.38% to 100%
< 26.75	84	75.32% to 90.57%	100	96.38% to 100%
< 27.25	87	78.8% to 92.89%	100	96.38% to 100%
< 27.75	91	83.6% to 95.8%	100	96.38% to 100%
< 28.25	92	84.84% to 96.48%	100	96.38% to 100%
< 29.25	93	86.11% to 97.14%	100	96.38% to 100%
< 30.25	95	88.72% to 98.36%	100	96.38% to 100%
< 30.75	97	91.48% to 99.38%	100	96.38% to 100%
< 35.5	98	92.96% to 99.76%	99	94.55% to 99.97%

Table S2: Sensitivity and specificity at different cut-off times for RT-LAMP.

Time cut-off (minutes)	Sensitivity (%)	95% CI	Specificity (%)	95% CI
< 11.5	4	1.1% to 9.926%	100	96.38% to 100%
< 12.5	15	8.645% to 23.53%	100	96.38% to 100%
< 13.25	20	12.67% to 29.18%	100	96.38% to 100%
< 13.75	23	15.17% to 32.49%	100	96.38% to 100%
< 14.25	34	24.82% to 44.15%	100	96.38% to 100%
< 14.75	39	29.4% to 49.27%	100	96.38% to 100%
< 15.25	46	35.98% to 56.26%	100	96.38% to 100%
< 15.75	48	37.9% to 58.22%	100	96.38% to 100%
< 16.25	54	43.74% to 64.02%	100	96.38% to 100%
< 16.75	57	46.71% to 66.86%	100	96.38% to 100%
< 17.25	63	52.76% to 72.44%	100	96.38% to 100%
< 17.75	65	54.82% to 74.27%	100	96.38% to 100%
< 18.25	71	61.07% to 79.64%	99	94.55% to 99.97%
< 18.75	75	65.34% to 83.12%	99	94.55% to 99.97%
< 19.5	81	71.93% to 88.16%	99	94.55% to 99.97%
< 20.25	87	78.8% to 92.89%	99	94.55% to 99.97%
< 21.25	88	79.98% to 93.64%	99	94.55% to 99.97%
< 22.25	89	81.17% to 94.38%	99	94.55% to 99.97%
< 23.5	89	81.17% to 94.38%	98	92.96% to 99.76%
< 24.75	90	82.38% to 95.1%	98	92.96% to 99.76%
< 25.25	90	82.38% to 95.1%	97	91.48% to 99.38%
< 25.75	91	83.6% to 95.8%	97	91.48% to 99.38%
< 26.5	92	84.84% to 96.48%	96	90.07% to 98.9%
< 27.25	93	86.11% to 97.14%	95	88.72% to 98.36%
< 27.75	94	87.4% to 97.77%	95	88.72% to 98.36%
< 29	95	88.72% to 98.36%	94	87.4% to 97.77%
< 30.5	95	88.72% to 98.36%	93	86.11% to 97.14%
< 31.25	95	88.72% to 98.36%	92	84.84% to 96.48%
< 31.75	95	88.72% to 98.36%	91	83.6% to 95.8%
< 32.25	95	88.72% to 98.36%	89	81.17% to 94.38%
< 32.75	95	88.72% to 98.36%	88	79.98% to 93.64%
< 33.5	96	90.07% to 98.9%	87	78.8% to 92.89%
< 34.5	96	90.07% to 98.9%	85	76.47% to 91.35%
< 35.5	96	90.07% to 98.9%	84	75.32% to 90.57%
< 36.5	96	90.07% to 98.9%	81	71.93% to 88.16%
< 37.5	96	90.07% to 98.9%	80	70.82% to 87.33%
< 38.5	96	90.07% to 98.9%	79	69.71% to 86.51%
< 39.5	96	90.07% to 98.9%	75	65.34% to 83.12%
< 40.25	96	90.07% to 98.9%	73	63.2% to 81.39%
< 41.25	96	90.07% to 98.9%	72	62.13% to 80.52%
< 42.25	96	90.07% to 98.9%	67	56.88% to 76.08%
< 42.75	96	90.07% to 98.9%	66	55.85% to 75.18%
< 43.75	96	90.07% to 98.9%	64	53.79% to 73.36%
< 44.75	96	90.07% to 98.9%	63	52.76% to 72.44%

Table S3: Sensitivity and specificity at different cut-off times for LAMP

Time cut-off (minutes)	Sensitivity (%)	95% CI	Specificity (%)	95% CI
< 11.25	1	0.02531% to 5.446%	100	96.38% to 100%
< 11.75	2	0.2431% to 7.038%	100	96.38% to 100%
< 12.25	4	1.1% to 9.926%	100	96.38% to 100%
< 12.75	7	2.861% to 13.89%	100	96.38% to 100%
< 13.25	12	6.357% to 20.02%	100	96.38% to 100%
< 13.75	19	11.84% to 28.07%	100	96.38% to 100%
< 14.25	25	16.88% to 34.66%	100	96.38% to 100%
< 14.75	34	24.82% to 44.15%	100	96.38% to 100%
< 15.25	43	33.14% to 53.29%	100	96.38% to 100%
< 15.75	47	36.94% to 57.24%	100	96.38% to 100%
< 16.25	51	40.8% to 61.14%	100	96.38% to 100%
< 16.75	53	42.76% to 63.06%	100	96.38% to 100%
< 17.25	56	45.72% to 65.92%	100	96.38% to 100%
< 17.75	58	47.71% to 67.8%	100	96.38% to 100%
< 18.5	60	49.72% to 69.67%	100	96.38% to 100%
< 19.25	63	52.76% to 72.44%	100	96.38% to 100%
< 19.75	64	53.79% to 73.36%	100	96.38% to 100%
< 20.25	67	56.88% to 76.08%	100	96.38% to 100%
< 20.75	72	62.13% to 80.52%	100	96.38% to 100%
< 21.25	74	64.27% to 82.26%	100	96.38% to 100%
< 21.75	76	66.43% to 83.98%	100	96.38% to 100%
< 22.5	77	67.51% to 84.83%	99	94.55% to 99.97%
< 23.25	78	68.61% to 85.67%	99	94.55% to 99.97%
< 23.75	81	71.93% to 88.16%	99	94.55% to 99.97%
< 24.25	82	73.05% to 88.97%	98	92.96% to 99.76%
< 24.75	86	77.63% to 92.13%	98	92.96% to 99.76%
< 25.25	88	79.98% to 93.64%	98	92.96% to 99.76%
< 26.75	92	84.84% to 96.48%	98	92.96% to 99.76%
< 28.25	92	84.84% to 96.48%	95	88.72% to 98.36%
< 28.75	93	86.11% to 97.14%	95	88.72% to 98.36%
< 29.25	93	86.11% to 97.14%	92	84.84% to 96.48%
< 29.75	95	88.72% to 98.36%	92	84.84% to 96.48%
< 30.5	96	90.07% to 98.9%	91	83.6% to 95.8%
< 31.25	96	90.07% to 98.9%	89	81.17% to 94.38%
< 31.75	97	91.48% to 99.38%	89	81.17% to 94.38%
< 32.5	97	91.48% to 99.38%	84	75.32% to 90.57%
< 33.5	97	91.48% to 99.38%	83	74.18% to 89.77%
< 34.25	97	91.48% to 99.38%	80	70.82% to 87.33%
< 34.75	97	91.48% to 99.38%	79	69.71% to 86.51%
< 35.25	97	91.48% to 99.38%	77	67.51% to 84.83%
< 35.75	97	91.48% to 99.38%	76	66.43% to 83.98%
< 36.5	97	91.48% to 99.38%	71	61.07% to 79.64%
< 37.5	97	91.48% to 99.38%	63	52.76% to 72.44%
< 38.5	97	91.48% to 99.38%	59	48.71% to 68.74%
< 39.5	97	91.48% to 99.38%	56	45.72% to 65.92%
< 40.25	97	91.48% to 99.38%	52	41.78% to 62.1%
< 40.75	97	91.48% to 99.38%	51	40.8% to 61.14%
< 41.5	98	92.96% to 99.76%	51	40.8% to 61.14%
< 43.5	98	92.96% to 99.76%	50	39.83% to 60.17%

Table S4. comparison between qPCR results and RT-LAMP on lateral flow devices for 40 clinical samples (n=4 technical replicates).

**RT-LAMP Lateral flow**	**qPCR** Ct (mean)	Ct (std)
positive	23.24	0.32
positive	24.08	0.07
positive	24.41	0.17
positive	24.83	0.11
positive	25.25	0.71
positive	25.83	0.38
positive	25.84	0.60
positive	26.17	0.01
positive	26.26	0.80
positive	26.39	0.36
positive	26.44	0.21
positive	26.56	0.04
positive	27.17	0.38
positive	28.71	0.23
negative	29.15	0.26
negative	32.13	0.23
negative	33.32	0.78
negative	-	
negative	-	
negative	-	
negative	-	
negative	-	
negative	-	
negative	-	
negative	-	
negative	-	
negative	-	
negative	-	
negative	-	
negative	-	
negative	-	
negative	-	
negative	-	
negative	-	
negative	-	
negative	-	
negative	-	
negative	-	
negative	-	
negative	-	
