# Supplementary material for: Paper microfluidic implementation of loop mediated isothermal amplification for early diagnosis of hepatitis C virus
Source: Nat Commun. 2021 Nov 30;12:6994. doi: 10.1038/s41467-021-27076-z (PMC8632961; doi:10.1038/s41467-021-27076-z)
Supplement: Supplementary file 3 — Reporting Summary [file 41467_2021_27076_MOESM3_ESM.pdf]

nature research

Corresponding author(s): Emma C. Thomson, Jonathan M. Cooper

Last updated by author(s): Sep 22 2021

Reporting Summary

Nature Research wishes to improve the reproducibility of the work that we publish. This form provides structure for consistency and transparency in reporting. For further information on Nature Research policies, see our Editorial Policies and the Editorial Policy Checklist.

Statistics

For all statistical analyses, confirm that the following items are present in the figure legend, table legend, main text, or Methods section.

n/a	Confirmed	
☐	☒	The exact sample size (*n*) for each experimental group/condition, given as a discrete number and unit of measurement
☐	☒	A statement on whether measurements were taken from distinct samples or whether the same sample was measured repeatedly
☐	☒	The statistical test(s) used AND whether they are one- or two-sided<br>*Only common tests should be described solely by name; describe more complex techniques in the Methods section.*
☐	☒	A description of all covariates tested
☐	☒	A description of any assumptions or corrections, such as tests of normality and adjustment for multiple comparisons
☐	☒	A full description of the statistical parameters including central tendency (e.g. means) or other basic estimates (e.g. regression coefficient) AND variation (e.g. standard deviation) or associated estimates of uncertainty (e.g. confidence intervals)
☐	☒	For null hypothesis testing, the test statistic (e.g. *F*, *t*, *r*) with confidence intervals, effect sizes, degrees of freedom and *P* value noted<br>*Give P values as exact values whenever suitable.*
☒	☐	For Bayesian analysis, information on the choice of priors and Markov chain Monte Carlo settings
☒	☐	For hierarchical and complex designs, identification of the appropriate level for tests and full reporting of outcomes
☒	☐	Estimates of effect sizes (e.g. Cohen's *d*, Pearson's *r*), indicating how they were calculated

Our web collection on statistics for biologists contains articles on many of the points above.

Software and code

Policy information about availability of computer code

Data collection	Microsoft Excel 2016.
Data analysis	Graphpad Prism Version 7, Microsoft PowerPoint 2016, Microsoft Excel 2016.

For manuscripts utilizing custom algorithms or software that are central to the research but not yet described in published literature, software must be made available to editors and reviewers. We strongly encourage code deposition in a community repository (e.g. GitHub). See the Nature Research guidelines for submitting code & software for further information.

Data

Policy information about availability of data

All manuscripts must include a data availability statement. This statement should provide the following information, where applicable:

- Accession codes, unique identifiers, or web links for publicly available datasets
- A list of figures that have associated raw data
- A description of any restrictions on data availability

The raw data, sequence alignment, gel blots and images that support the findings of this study are available in University of Glasgow's Enlighten: Research Data with the identifier: http://dx.doi.org/10.5525/gla.researchdata.1127.

Field-specific reporting

Life sciences study design

All studies must disclose on these points even when the disclosure is negative.

Sample size	A power calculation was performed for sensitivity of 90% (recommended minimal sensitivity for a point-of-care test for hepatitis C virus). Sensitivity of 90% is the null hypothesis (HO) and H1 is the alternative hypothesis. We assumed a 95% confidence and 80% power to detect a difference of 10% from presumption value of Sensitivity of 90%. This resulted in 86 samples from the equation. We had to accommodate for technical and procedural problems and possibility of contamination - we added an extra 15 % of samples. This resulted in a total number of 99 which was rounded up to 100 samples. Therefore, 100 HCV positive and 100 HCV negative samples were used in the study. For the second study on the performance of the lateral flow device, availability of samples did not allow to select such a large number and the study was not powered. sensitivity and specificity data are consequently not reported.
Data exclusions	No data were excluded from the analysis
Replication	Experiments were performed in duplicate and triplicate and were repeated at least two to three times. All experiments were successful.
Randomization	Samples from various genotypes and viral loads of hepatitis C virus were selected. Both hepatitis C virus positive and hepatitis C virus negative samples were included in the study. No randomization was required as all users were double blinded.
Blinding	Investigators were double-blinded during the data collection and data analysis of the clinical sensitivity and specificity.

Reporting for specific materials, systems and methods

We require information from authors about some types of materials, experimental systems and methods used in many studies. Here, indicate whether each material, system or method listed is relevant to your study. If you are not sure if a list item applies to your research, read the appropriate section before selecting a response.

Materials & experimental systems

n/a	Involved in the study	
☐	☒	Antibodies
☒	☐	Eukaryotic cell lines
☒	☐	Palaeontology and archaeology
☒	☐	Animals and other organisms
☐	☒	Human research participants
☒	☐	Clinical data
☒	☐	Dual use research of concern

Methods

n/a	Involved in the study	
☒	☐	ChIP-seq
☒	☐	Flow cytometry
☒	☐	MRI-based neuroimaging

Antibodies

Antibodies used	The antibodies used in the study are part of the lateral flow strips from Ustar, China.
Validation	not applicable

Human research participants

Policy information about studies involving human research participants

Population characteristics	Samples were obtained from 100 people with chronic hepatitis C virus infection and 100 hepatitis C virus negative people. A range of genotypes and viral loads were selected in the hepatitis C virus infected group. For the second study to characterise the performance of the lateral flow device, 40 samples were selected at random (20 positives and 20 negatives). Population characteristics including age, gender and treatment were not available as the samples were obtained from the Greater Glasgow Health Bio-repository.
Recruitment	There was no recruitment of participants as the patient samples were obtained through the Greater Glasgow Health Bio-repository
Ethics oversight	The Greater Glasgow Health Bio-repository and the NHS Research Ethics Committee (REC)

Note that full information on the approval of the study protocol must also be provided in the manuscript.
